# Genome-wide association study of vitamin D concentrations and bone mineral density in the African American-Diabetes Heart Study

**DOI:** 10.1371/journal.pone.0251423

**Published:** 2021-05-20

**Authors:** Nicholette D. Palmer, Lingyi Lu, Thomas C. Register, Leon Lenchik, J. Jeffrey Carr, Pamela J. Hicks, S. Carrie Smith, Jianzhao Xu, Latchezar Dimitrov, Jacob Keaton, Meijian Guan, Maggie C. Y. Ng, Yii-der I. Chen, Anthony J. Hanley, Corinne D. Engelman, Jill M. Norris, Carl D. Langefeld, Lynne E. Wagenknecht, Donald W. Bowden, Barry I. Freedman, Jasmin Divers

**Affiliations:** 1 Department of Biochemistry, Wake Forest School of Medicine, Winston-Salem, NC, United States of America; 2 Center for Precision Medicine, Wake Forest School of Medicine, Winston-Salem, NC, United States of America; 3 Division of Public Health Sciences, Wake Forest School of Medicine, Winston-Salem, NC, United States of America; 4 Department of Pathology, Wake Forest School of Medicine, Winston-Salem, NC, United States of America; 5 Department of Radiology, Wake Forest School of Medicine, Winston-Salem, NC, United States of America; 6 Department of Radiology, Vanderbilt University School of Medicine, Nashville, Tennessee, United States of America; 7 Molecular Genetics and Genomics Program, Wake Forest School of Medicine, Winston-Salem, NC, United States of America; 8 Department of Pediatrics, The Institute for Translational Genomics and Population Sciences, The Lundquist Institute for Biomedical Innovation at Harbor-UCLA Medical Center, Torrance, CA, United States of America; 9 Department of Nutritional Sciences, University of Toronto, Toronto, Ontario, Canada; 10 Department of Population Health Sciences, University of Wisconsin School of Medicine and Public Health, Madison, WI, United States of America; 11 Department of Epidemiology, Colorado School of Public Health, Aurora, CO, United States of America; 12 Department of Biostatistical Sciences, Wake Forest School of Medicine, Winston-Salem, NC, United States of America; 13 Department of Internal Medicine-Section on Nephrology, Wake Forest School of Medicine, Winston-Salem, NC, United States of America; Children’s Hospital of Philadelphia, UNITED STATES

## Abstract

Relative to European Americans, African Americans have lower 25-hydroxyvitamin D (25OHD) and vitamin D binding protein (VDBP) concentrations, higher 1,25-dihydroxyvitamin D (1,25(OH)_2_D_3_) concentrations and bone mineral density (BMD), and paradoxically reduced burdens of calcified atherosclerotic plaque (subclinical atherosclerosis). To identify genetic factors contributing to vitamin D and BMD measures, association analysis of >14M variants was conducted in a maximum of 697 African American-Diabetes Heart Study participants with type 2 diabetes (T2D). The most significant association signals were detected for VDBP on chromosome 4; variants rs7041 (β = 0.44, SE = 0.019, P = 9.4x10^-86^) and rs4588 (β = 0.17, SE = 0.021, P = 3.5x10^-08^) in the group-specific component (vitamin D binding protein) gene (*GC*). These variants were found to be independently associated. In addition, rs7041 was also associated with bioavailable vitamin D (BAVD; β = 0.16, SE = 0.02, P = 3.3x10^-19^). Six rare variants were significantly associated with 25OHD, including a non-synonymous variant in *HSPG2* (rs116788687; β = -1.07, SE = 0.17, P = 2.2x10^-10^) and an intronic variant in *TNIK* (rs143555701; β = -1.01, SE = 0.18, P = 9.0x10^-10^), both biologically related to bone development. Variants associated with 25OHD failed to replicate in African Americans from the Insulin Resistance Atherosclerosis Family Study (IRASFS). Evaluation of vitamin D metabolism and bone mineral density phenotypes in an African American population enriched for T2D could provide insight into ethnic specific differences in vitamin D metabolism and bone mineral density.

## Introduction

T2D and osteoporosis are diseases of aging which contribute to increased fracture risk [1]. Related to vitamin D metabolism, serum concentrations of 25-hydroxyvitamin D (25OHD), 1,25-dihydroxyvitamin D (1,25(OH)_2_D_3_), vitamin D binding protein (VDBP), and intact parathyroid hormone (iPTH) differ markedly between individuals of African and European ancestry [2–6]; however, bioavailable vitamin D (BAVD) levels appear to be similar. In concert with differences in vitamin D metabolism, African (versus European) ancestry is associated with higher bone mineral density (BMD) and lower prevalence of osteoporosis [7–9]. The effects of 25OHD, 1,25(OH)_2_D_3_ and iPTH on their target organs/cells may also differ based on race/ethnicity. For example, skeletal resistance to the effects of iPTH is more pronounced in individuals who possess recent African ancestry [10].

Beyond population ancestry-based differences in bone mineral density and vitamin D metabolism, calcified atherosclerotic plaque [11] and calcium-containing kidney stones [12] also develop significantly less often in African Americans than European Americans. Inverse relationships exist between the development and progression of calcified atherosclerotic plaque (*e*.*g*., subclinical atherosclerosis) with bone mineralization; this relationship is independent from ethnicity [13]. Literature evidence supports higher risks for development of subclinical atherosclerosis and osteoporosis in populations of European ancestry [14].

The African American-Diabetes Heart Study (AA-DHS) was designed to investigate biologic relationships between vitamin D and its metabolites, subclinical atherosclerosis, and skeletal health. Unique aspects of this type 2 diabetes (T2D)-affected African American cohort is their generally preserved kidney function, low levels of albuminuria, and well-treated blood pressures, serum lipids, and glucose levels suggesting adequate access to healthcare. Herein, we report results of genome-wide association studies (GWAS) for concentrations of 25OHD, 1,25(OH)_2_D_3_, VDBP, BAVD and iPTH and the traits of volumetric BMD (vBMD) determined by quantitative computed tomography (QCT) in the thoracic and lumbar spine in AA-DHS participants.

## Materials and methods

### Study participants

AA-DHS includes African Americans with T2D recruited from two Wake Forest School of Medicine (WFSM) studies: the family-based Diabetes Heart Study (DHS) and unrelated individuals in the AA-DHS. DHS is a cross-sectional study of European American and African American families with siblings concordant for T2D. AA-DHS started after DHS and enrolled unrelated African Americans using identical inclusion criteria. AA-DHS objectives are to improve understanding of ethnic differences in coronary artery calcification and calcified plaque in populations of African and European ancestry. T2D was diagnosed in all participants after the age of 30 years in the absence of diabetic ketoacidosis and defined as fasting blood glucose ≥126 mg/dL or a random glucose ≥200 mg/dL, history of physician diagnosis of diabetes, or use of insulin or an oral hypoglycemic agent. The study was approved by the WFSM Institutional Review Board (BG05-033) and all participants provided written informed consent.

### Phenotypes

25OHD was measured by Quest Diagnostics Nichols Institute (San Juan Capistrano, CA) and LabCorp using liquid chromatography and mass spectrometry and an immunochemiluminometric assay performed on the DiaSorin LIASON® instrument, respectively, with a high paired sample concordance (n = 14, r^2^ = 0.92). 1,25(OH)_2_D_3_ was measured at Quest or LabCorp® using liquid chromatography mass spectrometry and column chromatography, radioimmunoassay, respectively. iPTH was measured at LabCorp (Burlington, NC) using an electrochemiluminescence immunoassay.

VDBP was measured in EDTA plasma samples that had been continuously stored at -80°C without thawing since collection at baseline visits. Frozen plasma was thawed in a 37°C water bath for 15 minutes, placed on ice, and then centrifuged at 1700 x g (2800 rpm) for 30 minutes at 4°C. VDBP was determined using a Quantikine® Human Vitamin D BP ELISA (cat. no. DDBP0; R&D Systems; Minneapolis, MN) according to manufacturer’s instructions. Intra-assay and inter-assay coefficients of variation were <10%. All assays were performed using a single lot of reagents and calibrators at WFSM. BAVD was computed as described by Powe et al. [6] using dissociation constants for the VDBP genotype variants as described by Arnaud and Constans [15].

Trabecular vertebral volumetric BMD (vBMD in mg/cm3) in the thoracic spine (T8-T11) and lumbar spine (T12-L3) were measured during computed tomography (CT) examination of the chest and abdomen using a standardized scanning protocol as previously described [16].

### Genotyping

DNA from AA-DHS participants was extracted from peripheral blood using the PureGene system (Gentra Systems, Minneapolis, MN). The AA-DHS GWAS utilized the Illumina 5M chip. Quality control (QC) checks in AA-DHS led to the exclusion of 12 individuals from the analyses: six had call rates <95%, two had discordant self-reported and genetically determined sex, one had a heterozygosity score outside of the mean ±4 times the standard error interval, two had the same sample identifiers and one had 100% European ancestry. GWAS analyses were performed on up to 697 individuals.

Imputation was performed using IMPUTE2 with phased haplotype data obtained from SHAPEIT2 [16]. Directly genotyped variants with a Hardy-Weinberg Equilibrium (HWE) p-value ≥1x10^-04^ and a call rate ≥95% were included and imputation was based on 3,436,913 autosomal variants. The multi-ethnic 1,000 Genomes Phase I integrated variant set release (v3) was used as the reference panel. Statistical analysis was performed for imputed variants that had an info score above 50%, a minor allele frequency (MAF) >1% and a HWE p-value ≥1x10^-04^.

### Statistical analysis

Analyses were run using 25OHD, 1,25(OH)_2_D_3_, VDBP, BAVD, iPTH, thoracic vBMD, and lumbar vBMD as continuous traits. Phenotype correlations were explored using Spearman correlation coefficients. Phenotypes were transformed to best approximate a normal distribution, i.e. square root of 1,25(OH)_2_D_3_ plus 1, thoracic vBMD, and lumbar vBMD; natural log of 25OHD plus 1 and iPTH; and log BAVD and VDBP. Age, sex, body mass index (BMI), kidney function based on the Chronic Kidney Disease-Epidemiology Collaboration (CKD-EPI) estimated glomerular filtration rate (eGFR), multivitamin use, and global African ancestry proportions were included as covariates. Global African ancestry proportion estimates were obtained by averaging the local ancestry estimates across the genome obtained from LAMP-ANC and HAPMIX [17,18]. This approach utilized a linkage disequilibrium (LD) pruning algorithm that was applied with an r^2^ threshold of 0.8 to select a subset of variants. Observed data at these variants were then combined with HapMap phase 3 genotypes obtained from Yoruba and CEPH samples; the HapMap samples were used as anchoring populations and were not included in the analysis. Results obtained from LAMP-ANC and HAPMIX were comparable, i.e. Spearman correlation estimates ranged between 0.88 and 0.97 for the 22 autosomes. For these continuous outcomes, linear mixed models were fitted using the Genome-wide Efficient Mixed Model Analysis (GEMMA) software [10] and each variant was tested for association using an additive model. To account for inter-assay variability for 25OHD and 1,25(OH)_2_D_3,_ results from the individual platforms were combined via meta-analysis. Genome-wide significance was defined as a p-value <5.0x10^-8^. In addition, a principal components analysis (PCA) was used to determine the number of phenotypes needed to explain 95% of the observed variation. This number was then used to establish a more conservative threshold for significance. For statistically significant variants, a secondary analysis was performed with the inclusion of sex as a three level variable to control for menopause status. Conditional analysis for significantly associated variants was performed with inclusion of variant genotypes as covariates in the analysis, i.e. VDBP and BAVD. For variants rs7041 and rs4588, interaction was tested in a model which included both variants as main effects and a centered interaction term with adjustment for the same set of covariates as the main effect only models. In addition to the discovery analysis, previously reported variants associated with 25OHD [19], VDBP [20], iPTH [21] and lumbar vBMD [22] were examined in their respective AA-DHS GWAS.

### Replication

Replication of associations observed for 25OHD and 1,25(OH)_2_D_3_ was performed among Insulin Resistance Atherosclerosis Family Study (IRASFS) African Americans [23]. IRASFS is a population-based cohort in which to evaluate generalizability of findings from a diabetes-enriched population. IRASFS African Americans were recruited from Los Angeles, CA with race/ethnicity determined by self-report. Informed consent was obtained from all participants.

Levels of 25OHD and 1,25(OH)_2_D_3_ were measured as previously described [24]. Samples (n = 554) were genotyped on the Multi-Ethnic Genotyping Array (MEGA; Illumina, San Diego, CA) [25]. A total of 1,705,970 variants were successfully called for QC and analysis. Sample QC was performed to remove individuals with low call rates (<0.95), sex discordance, DNA contamination, or non-African ancestry. Duplicate samples were compared, and one of each duplicate pair was removed. Variants with missing position or alleles, allele mismatch, call rates <95%, departure from HWE (P≤10^−04^), frequency difference >0.2 compared with the 1000 Genomes Project phase 3 reference panel, and monomorphic variants were removed. Variants and samples that passed QC were used to perform pre-phasing with SHAPEIT2 [26] and imputation with IMPUTE2 [27] using a combined haplotype reference panel from the 1000 Genomes Project phase 3 (1000 Genomes Project Consortium 2010) and the African Genome Variation Project (AGVP) [28]. Variants with imputation info scores >0.4 were included in analysis. Tests of association between the seven genetic variants associated with 25OHD and 1,25(OH)_2_D_3_ were computed using the Wald test from the variance component model implemented in Sequential Oligogenic Linkage Analysis Routines (SOLAR) [29]. Phenotypes were transformed to best approximate the distributional assumptions of conditional normality and homogeneity of variance, i.e. both were square root transformed. Admixture estimates were calculated using maximum likelihood estimation of individual ancestries as implemented in ADMIXTURE [30]. Genetic associations were calculated adjusting for age, sex, and admixture estimates. The primary inference was the additive model.

## Results

Demographic and clinical characteristics of AA-DHS participants are presented in **Table 1**. The majority of participants were female (58.1%), with mean ± SD age 56.3 ± 9.6 years, BMI 35.2 ± 8.5 kg/m^2^ and 70% African ancestry as estimated from the Omni5 array. Participants had long-standing diabetes (10.3 ± 8.0 years) that was relatively well-controlled (HbA1c of 8.2 ± 2.2%). Mean 25OHD was 20.4 ± 11.8 ng/mL, 1,25(OH)_2_D_3_ 46.7 ± 17.1 pg/mL and VDBP 81.2 ± 68.5 ug/mL. iPTH 54.3 ± 29.3 pg/mL and thoracic vBMD and lumbar vBMD 198.1 ± 53.5 and 174.0 ± 47.2 mg/cm^3^, respectively. Phenotypic correlations are presented in **S1 Table.**

**Table 1:** Demographic, laboratory, and imaging characteristics of the African American-Diabetes Heart Study cohort.

Variables	Men (n_max_ = 292)		Women (n_max_ = 405)		Combined (n_max_ = 697)	
	Mean	SD	Mean	SD	Mean	SD
Age (years)	56.5	9.6	56.1	9.6	56.3	9.6
Body Mass Index (kg/m^2^)	32.3	7.2	37.2	8.8	35.2	8.5
African Ancestry proportion (%)	0.70	0.20	0.80	0.2	0.70	0.20
25-hydroxyvitamin D (ng/mL)	20.0	10.4	20.8	12.7	20.4	11.8
1,25-dihydroxyvitamin D (pg/mL)	45.2	17.0	47.8	17.2	46.7	17.1
Vitamin D binding protein (μg/mL)	89.1	76.6	75.0	60.8	81.2	68.5
Intact parathyroid hormone (pg/mL)	49.7	26.1	57.8	31.1	54.3	29.3
Thoracic vBMD (mg/cm^3^)	194.5	50.5	200.6	55.5	198.1	53.5
Lumbar vBMD (mg/cm^3^)	173.8	44.1	174.1	49.4	174.0	47.2
Diabetes duration (years)	10.7	8.6	10.1	7.5	10.3	8.0
HbA1c (%)	8.2	2.0	8.2	2.3	8.2	2.2
Glucose (mg/dl)	158.4	75.9	148.3	63.8	152.5	69.3
C-reactive protein (mg/dl)	0.70	1.0	1.1	1.7	0.9	1.5
Low density lipoprotein cholesterol (mg/dl)	104.0	38.7	110.1	37.0	107.5	37.8
High density lipoprotein cholesterol (mg/dl)	44.0	12.9	50.1	14.1	47.6	13.9
Triglycerides (mg/dl)	134.7	156.6	125.4	86.4	129.3	120.8
ACE inhibitor use (%)	44.5		39.8		41.8	
Current smoker (%)	31.3		20.3		24.9	
Past smoker (%)	40.4		32.6		35.9	
Hypertension (%)	81.3		85.3		83.6	
Lipid-lowering medication (%)	47.8		50		49.1	

Abbreviations: ACE, angiotensin converting enzyme inhibitor; BMI, body mass index; HbA1c, hemoglobin A1c; LDL, low density lipoprotein; HDL, high density lipoprotein; SD, standard deviation; vBMD, volumetric bone mineral density.

**Fig 1:**
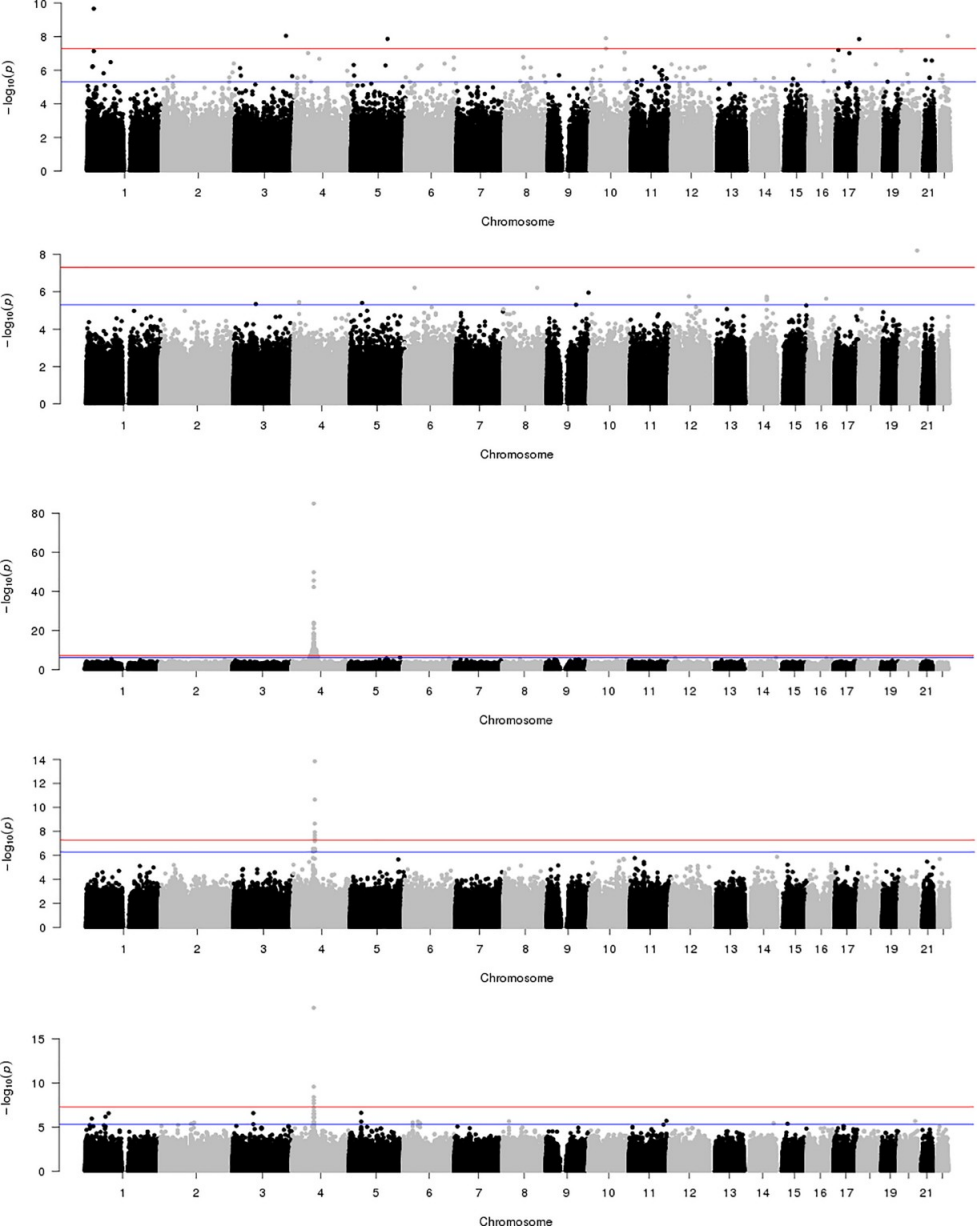
Manhattan plots for trait association results in the African American-Diabetes Heart Study (AA-DHS). A. 25-hydroxyvitamin D, B. 1,25-dihydroxyvitamin D, C. Vitamin D Binding Protein, D. Vitamin D Binding Protein adjusted for rs7041 and E. Bioavailable Vitamin D.

**Fig 2:**
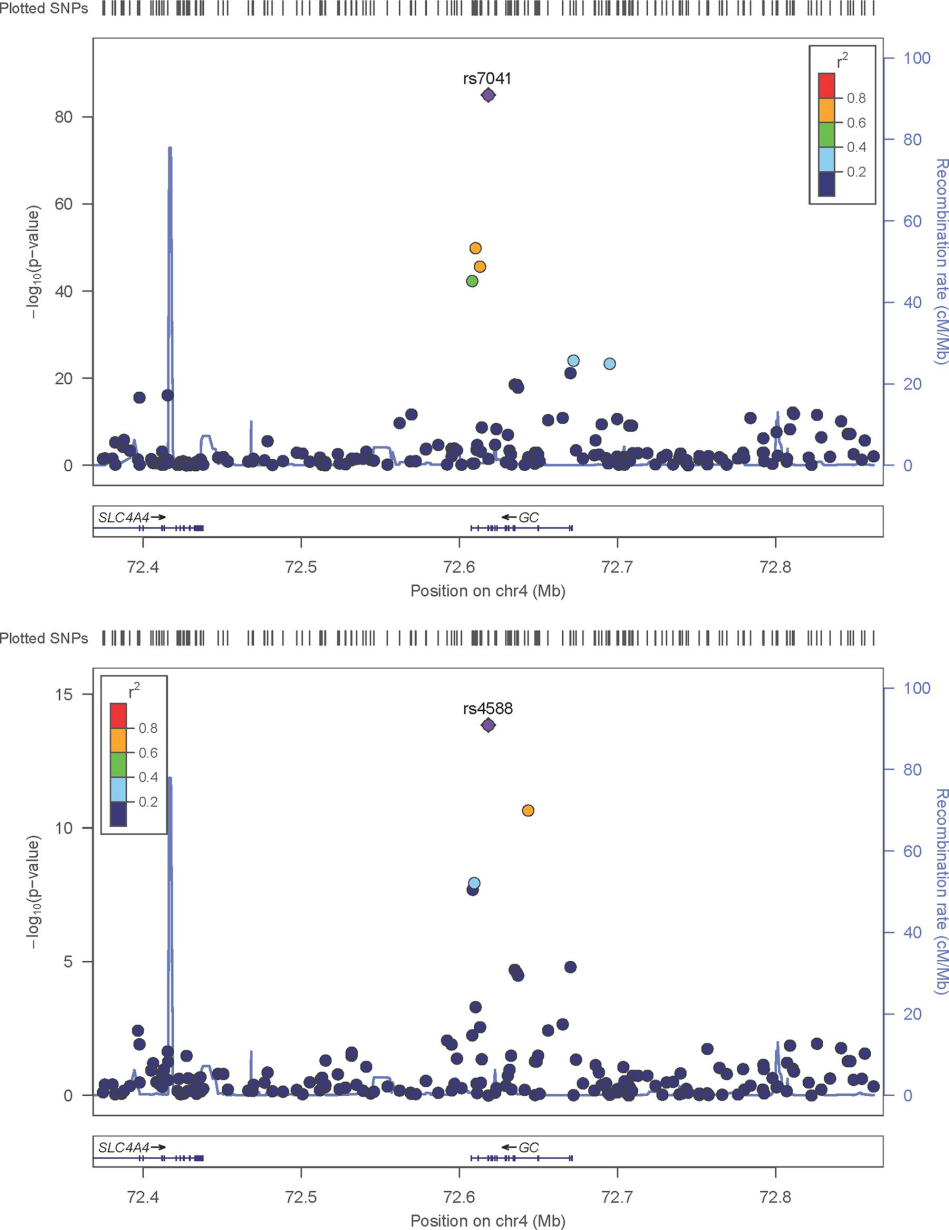
Regional association plots for variants associated at genome-wide significance (P<5.0x10^-8^) with VDBP in the AA-DHS cohort. The -log10 (p-value) is shown on the left y-axis, recombination rates [expressed in centiMorgans (cM) per Mb; NCBI Build GRCh37; highlighted in blue] are shown on the right y-axis and position in Mb is on the x-axis. Pairwise linkage disequilibrium (r^2^) of each variant with the top variant in the region is indicated by its color. A. rs7041 (VDBP) and B. rs4588 (VDBP, adjusted for rs7041).

GWAS analyses were conducted for traits 25OHD, 1,25(OH)_2_D_3_, VDBP, BAVD, iPTH, thoracic vBMD, and lumbar vBMD. An inflation factor (λ) less than 1.03 in all analyses suggests that population stratification was adequately controlled. Genome-wide significant results (P<5.0x10^-08^) are presented in **Table 2, Fig 1**. In addition, PCA revealed that five of the seven phenotypes are needed to explain 95% of the observed variation, suggesting a more stringent threshold of 1.0x10^-8^ would account for the number of phenotypes examined (**S2 Table)**. The most significant association signal detected was for VDBP on chromosome 4. In total, 172 variants were significantly associated (P<5.0x10^-08^) with VDBP across a 20Mb interval with the most significant signal observed at rs7041 (β = 0.44, SE = 0.019, P = 9.4x10^-86^; **Fig 2**) in the group-specific component (VDBP) gene (*GC*). Analysis conditioning on genotypes at rs7041 revealed six variants on chromosome 4 emerging as statistically significant (P = 4.1x10^-06^–0.37 in the additive model versus P = 1.4x10^-14^–3.5x10^-08^ in an rs7041-adjusted model). These variants ranged in correlation (r^2^ = 0.00–0.99) but were uncorrelated with the index variant (rs7041; r^2^<0.02). The most significant signal after conditional analysis was rs4588 which was not correlated with rs7041 (r^2^ = 0.02) suggesting a second independent signal at the *GC* locus (**Fig 2**). Analysis conditioned on genotypes at variants rs7041 and rs4588 abolished association of additional variants (P>1.1x10^-07^).

**Table 2:** Summary of variants associated at genome-wide significance (P<5.0x10^-8^) with vitamin D concentrations, parathyroid hormone concentrations, and bone mineral density in the African American-Diabetes Heart Study cohort.

Variant	Position (hg19)	Alleles^1^	MAF^2^	Gene^3^	Beta ± SE	Additive P-value^4^
***25-hydroxyvitamin D***						
rs116788687	chr1:22181360	G/C	0.0079	*HSPG2*	-1.07 ± 0.17	**2.17x10**^**-10**^
rs143555701	chr3:171114695	T/G	0.0066	*TNIK*	-1.01 ± 0.18	**8.98x10**^**-09**^
rs116950775	chr22:44764343	T/C	0.0079	*KIAA1644/LDOC1L*	-1.04 ± 0.18	**9.29x10**^**-09**^
rs114001906	chr10:51799688	C/T	0.0033	*FLJ31813*	-1.06 ± 0.19	1.25x10^-08^
rs111955953	chr5:121180672	C/A	0.014	*-/FTMT*	-1.06 ± 0.19	1.36x10^-08^
rs117075918^5^	chr17:77990613	C/T	0.0099	*TBC1D16*	-1.23 ± 0.22	1.40x10^-08^
***1*,*25-dihydroxyvitamin D***						
rs80068476	chr20:56603691	T/C	0.011	*-/C20orf85*	-2.54 ± 0.44	**6.19x10**^**-09**^
***Vitamin D Binding Protein***						
rs7041	chr4:72618334	C/A	0.16	*GC*	0.44 ± 0.019	**9.35x10**^**-86**^
***Vitamin D Binding Protein (adjusted for GC rs7041)***						
rs4588	chr4:72618323	T/G	0.11	*GC*	0.17 ± 0.021	**1.43x10**^**-14**^
rs1155563	chr4:72643488	C/T	0.12	*GC*	0.13 ± 0.019	**2.22x10**^**-11**^
rs221999^5^	chr4:72649048	G/A	0.12	*GC*	0.13 ± 0.021	**2.23x10**^**-09**^
rs3755967	chr4:72609398	T/C	0.096	*GC*	0.14 ± 0.023	1.15x10^-08^
rs2282679	chr4:72608383	G/T	0.095	*GC*	0.13 ± 0.024	2.03x10^-08^
rs9016^5^	chr4:72618296	T/C	0.023	*GC*	-0.26 ± 0.046	3.52x10^-08^
***Bioavailable Vitamin D***						
rs7041	chr4:72618334	C/A	0.16	*GC*	0.16 ± 0.02	**3.30x10**^**-19**^
rs222047	chr4:72610208	C/A	0.19	*GC*	0.11 ± 0.02	**2.68x10**^**-10**^
rs705119	chr4:72613036	C/A	0.15	*GC*	0.12 ± 0.02	**4.09x10**^**-09**^
rs705117	chr4:72608115	T/C	0.28	*GC*	-0.09 ± 0.02	**9.67x10**^**-09**^
rs11939173	chr4:72672158	A/G	0.15	*GC/NPFFR2*	-0.11 ± 0.02	2.05x10^-08^

^1^Reference/other allele

^2^minor allele frequency

^3^nearest annotated gene within 500kb

^4^Values in bold were remained significant after correcting for multiple correlated traits (n = 5)

^5^Variants were imputed from 1000 Genomes.

To model the joint effects of variants rs7041 and rs4588 on VDBP, a model that included both variants as main effects and a centered interaction term was examined. Mean VDBP levels by genotype combination at rs7041 and rs4588 are presented in **S3 Table**. The parameter estimate associated with the interaction term was nominally significant (-0.13 ± 0.06, P = 0.028), suggesting that the joint effect of risk variants at the two variants was not linear.

Similarly, five variants in or near the GC locus were associated with BAVD (**Table 2**). These variants were moderately correlated (r^2^ = 0.15–0.76) and ranged in association from P = 3.3x10^-19^ to 2.1x10^-08^. Analysis conditioned on genotypes at variant rs7041 abolished association of additional variants (P = 0.013–0.85). Further examination of analyses conditional on the genotypes at variant rs7041 revealed only nominal evidence of association genome-wide (P>3.3x10^-07^).

Six variants were significantly associated with 25OHD (P = 2.2x10^-10^–1.4x10^-08^, **Table 2**). Adjustment for seasonal changes in 25OHD and dietary use of vitamin supplements did not significantly impact these observations. The most significant signal, rs116788687, was a non-synonymous variant which resulted in the substitution of a methionine for isoleucine in the heparan sulfate proteoglycan 2 gene (*HSPG2*). This is a rare coding mutation (MAF = 0.0079%) that was observed in ten heterozygotes and one homozygote. Presence of the G allele resulted in a decrease of 25OHD (16.8 vs 20.5 ng/mL). Additionally, variant rs143555701 in the TRAF2 and NCK interacting kinase gene (*TNIK*) was associated with 25OHD (P = 9.0x10^-09^). The minor allele of this rare variant was associated with a decrease in 25OHD levels. Moreover, variant rs117075918 was associated with 25OHD (P = 1.4x10^-08^). This intronic variant had a MAF of 0.99% and the minor allele (G) was associated with a decrease in 25OHD levels. The remaining three variants associated with 25OHD were intergenic (rs116950775, P = 9.3x10^-09^ and rs111955953, P = 1.4x10^-08^) or in a hypothetical gene region (rs114001906, P = 1.3x10^-08^).

A single variant was significantly associated with 1,25(OH)_2_D_3_ in the cohort. Variant rs80068476 (MAF = 1.1%) was associated with a decrease in 1,25(OH)_2_D_3_ levels (P = 6.2x10^-09^). This variant is located intergenically on chromosome 20q13.32, 317kb distal to the prostate transmembrane protein, androgen induced 1 gene (*PMEPA1*) and 122kb proximally to the chromosome 20 open reading frame 85 gene (*C20orf85*). No variants were identified for association iPTH, thoracic vBMD, or lumbar vBMD that survived multiple comparison correction (P<5.0x10^-08^, **S1 Fig**).

Results from previously reported genome-wide significant loci associated with 25OHD [19], VDBP [20], iPTH [21] and lumbar vBMD [22] are presented in **S4 Table**.

Although few studies have targeted recruitment of African Americans with T2D and measures of vitamin D, replication was attempted in the IRASFS African American cohort. In total, six variants were evaluated for replication with 25OHD and one variant was evaluated for replication with 1,25(OH)_2_D_3_. Consistent with the allele frequencies observed in AA-DHS, all variants were low-frequency, i.e. MAF<5%. Although no variant was identified with nominal evidence of association (P<0.05), a consistent direction of effect was observed for rs143555701, rs114001906, and rs117075918 with 25OHD and rs80068476 with 1,25(OH)_2_D_3_ (**S5 Table**).

## Discussion

A GWAS was performed for key clinical phenotypes targeting vitamin D metabolism and bone mineral density in an African American cohort with T2D. Ten independent genome-wide significant loci were identified. Of these phenotypes, seven rare variants were significantly associated with 25OHD or 1,25(OH)_2_D_3_ consistently resulting in a decrease of their circulating levels. In addition, a detailed analysis of VDBP not only confirmed the striking association of a non-synonymous mutation in *GC*, but also provided evidence for a second association signal at this locus.

The most significant association signal observed from this series of GWAS was association of a non-synonymous variant, rs7041, in exon 11 of *GC* with VDBP (P = 9.35x10^-86^). *GC* encodes the VDBP which is the primary vitamin D carrier protein which regulates the bioavailability of 25OHD. In contrast to the seminal GWAS publications [20,31] performed in European-derived populations where the T allele frequency was 35%, in AA-DHS the frequency was 16%. Despite this difference, rs7041 was associated with a 4.5-fold reduction in VDBP, i.e. 271.9, 149.4, and 49.9 ug/mL for 0, 1, and 2 copies of rs7041-A, respectively, consistent with prior results in a European-derived population [20], but with approximately two fold lower levels of VDBP by genotype in this African American population with T2D. Despite the fact that vitamin D level is an important determinant of BMD [32], this variant was not significantly associated with the measures of vBMD assessed herein, i.e. lumbar vBMD, P = 0.12 or thoracic vBMD, P = 0.04. Conditional on association at rs7041, we observed a second independent signal of association at the *GC* locus. A second non-synonymous variant, rs4588, also located in *GC* was associated with VDBP (P = 1.4x10^-14^). Notably, this variant was only nominally associated with VDBP (P = 0.031) prior to conditional analysis. This mutation was initially reported by immunophelometric methods for the quantification of the three most common VDBP isotypes [31]. In AA-DHS the minor allele rs4588-T (MAF = 11%) was associated with an increase in VDBP, i.e. 80.7, 81.9, and 100.2 ug/mL for 0, 1, and 2 copies, respectively. An interaction model including the effects of rs7041 and rs4588 was evaluated with VDBP as the outcome. Although not previously evaluated, we posited that variant lowering alleles would interact and result in reductions in VDBP. Upon formal analysis and consistent with the single variant results, the interaction model was associated with decreased VDBP levels (P = 0.028).

Consistent with its role as the primary vitamin D carrier protein, variants at the *GC* locus were also associated with BAVD in the AA-DHS cohort. The majority of 25OHD in circulation is bound to carrier protein, i.e. VDBP (85–90%) and albumin (10–15%) [33], leaving less than 1% of 25OHD free and available to cells (BAVD). The most significant variant associated with BAVD was the non-synonymous variant, rs7041 (P = 3.3x10^-19^), associated with a decrease in BAVD, i.e. 6.8, 4.4, and 3.0 ng/mL for 0, 1, and 2 copies of rs7041-C, respectively. In total, five variants were significantly associated with BAVD at the GC locus; however, association was abolished after analyses conditioned on rs7041. Interestingly, variant rs4588 was not associated with BAVD (P = 0.68) or after analysis conditioned on rs7041 (P = 0.19). Taken together, these findings represent the first GWAS for BAVD in the African American population and compliment previously published findings in a European American population [20].

Six loci were significantly associated with 25OHD. The most significant observation was a non-synonymous variant, rs116788687 (P = 2.2x10^-10^) in *HSPG2*. *HSPG2* encodes a ubiquitous heparan sulfate proteoglycan, perlecan. Perlecan is a large multidomain proteoglycan involved in diverse biological functions. In knockout mouse models, perlecan has been identified as a major component of the pericellular material surrounding osteocyte processes where it functions to maintain structural integrity which is crucial for uninhibited interstitial fluid movement [34]. This variant was not associated in IRASFS African Americans (P = 0.92), although few had T2D (n = 76).

Intronic variant rs143555701 in *TNIK*, located on chromosome 3, was associated with decreased 25OHD levels (P = 9.0x10^-10^). *TNIK* encodes a serine/threonine kinase that functions as an activator of the Wnt signaling pathway [35]. Both vitamin D, specifically 1,25(OH)_2_D_3_, and the Wnt signaling pathway have been implicated in osteoblastic proliferation and differentiation [36]. Despite this relationship, this variant was not associated with variation in 1,25(OH)_2_D_3_ levels (P = 0.53). Based on these observations, the *TNIK* locus could represent a cross talk pathway between vitamin D and the Wnt signaling pathway. A consistent direction of effect was observed in IRASFS, whereby increasing copies of the minor allele, rs143555701-T, were associated with decreases in 25OHD although this finding was nonsignificant (P = 0.45). Additional loci significantly associated with 25OHD were located intergenically (rs116950775, P = 9.3x10^-09^ and rs111955953, P = 1.4x10^-08^) or in genes of relatively unknown function (rs114001906, P = 1.3x10^-08^ and rs117075918, P = 1.4x10^-08^). While all associated variants were directly genotyped in AA-DHS, suggesting high quality genotype data, none were replicated in IRASFS.

The largest GWAS meta-analysis of circulating 25OHD conducted to date was from the Study of Underlying Genetic Determinants of Vitamin D and Highly Related Traits (SUNLIGHT) Consortium which included 33,996 European ancestry participants from 15 cohorts [37]. In total, four loci were significantly associated with 25OHD. The most significant signal observed was rs2282679 in the *GC* locus which was not associated with 25OHD in AA-DHS (P = 0.13). Notably, rs2282679 and rs4588 are in complete LD (D’/r^2^ = 1.0) in European ancestry populations but not in African-derived populations (YRI, D’ = 0.40, r^2^ = 0.11), i.e. rs4588 but not rs2282679 was associated with 25OHD in AA-DHS. Index variants for the remaining three loci were not directly assessed in AA-DHS; however, regional examination of *DHCR7* (P>0.17), *CYP2R1* (P>0.098) and *CYP24A1* (P>0.028) failed to identify signals of association. These results are not surprising given the large difference in sample size and differential recruitment strategies. Specifically, AA-DHS focused on individuals with recent African ancestry who, on average, have lower levels of 25OHD levels than European populations, i.e. 20.4 ng/mL in AA-DHS vs 27.92 ng/mL in SUNLIGHT European-derived populations. Moreover, AA-DHS is enriched for T2D which is associated with altered vitamin D homeostasis, i.e. low 25OHD [38]. These observations support the potential for a differential genetic basis between studies.

Circulating vitamin D is hydroxylated in the kidney to form biologically active 1,25(OH)_2_D_3_ [39]. In contrast to the multiple genetic associations observed with 25OHD, only a single variant was significantly associated with 1,25(OH)_2_D_3_. Variant rs80068476 (P = 6.2x10^-09^) was associated with decreased 1,25(OH)_2_D_3_ levels. This variant is an intergenic variant located between microRNA 4532 (*MIR4532*), a non-coding RNA involved in post-transcriptional regulation of gene expression, and protein phosphatase 4, regulatory subunit 1-like (*PPP4R1L*). This variant was not associated in IRASFS African Americans (P = 0.35) but had a consistent direction of effect whereby increasing numbers of the minor allele rs80068476-T were associated with decreasing levels of 1,25(OH)_2_D_3_. In comparison with prior studies, only one GWAS for 1,25(OH)_2_D_3_ has been published. In a Hispanic American sample from the IRASFS, a single variant, rs6680429, in the Dab, reelin signal transducer, homolog 1 gene (*DAB1*) was associated in a pilot study of 229 individuals with replication in the total cohort (n = 1190) [40]. This variant was not associated in the AA-DHS cohort (P = 0.64), despite being a relatively common genetic variant in AA-DHS African Americans (MAF = 0.31).

In addition to the discovery analysis, previously reported variants associated with 25OHD [19], VDBP [20], iPTH [21] and lumbar vBMD [22] were examined in AA-DHS for association (**S4 Table**). The most significant variants which replicated published associations were with VDBP, i.e. rs7041, P = 9.35x10^-86^ and rs705117, P = 4.58x10^-43^. In addition, three variants previously associated with lumbar vBMD (rs344081, P = 0.021; rs9466056, P = 0.016 and rs6959212, P = 0.030) and one variant previously associated with iPTH [21] (rs6127099, P = 0.049 and rs219779, P = 0.012) were nominally significant and had a consistent direction of effect in AA-DHS. There were no variants, previously associated with 25OHD [19], found to be significant in AA-DHS. This is not surprising as the SUNLIGHT Consortium was a large meta-analysis (n = 79,366) of European-derived populations.

GWAS remains a powerful tool to understand the genetic architecture of complex biological phenotypes, but it is not without limitations. While the sample size associated with contemporary GWAS, e.g. type 2 diabetes, blood lipids, blood pressure, etc., are comparatively large, the vitamin D and BMD phenotypes examined here represent traits that are not routinely assessed. Moreover, the study design for the AA-DHS focuses exclusively on African Americans with T2D; thus, limiting our ability to identify an identically ascertained replication cohort. In addition, this is among the first genetics studies focused exclusively on the African American population where differences in bone mineral density and the vitamin D metabolism often oppose those in European-derived populations. Despite identification of coding variants related to vitamin D metabolism, additional loci located in intronic and intergenic regions challenges our ability to make causal inferences. This suggests potential contributions from variants not currently assessed, despite use of a dense genotyping array and imputation to 1000 Genomes.

In conclusion, a GWAS of phenotypes focused on vitamin D metabolism and bone mineral density was conducted in the AA-DHS cohort. Recruited on the basis of T2D, the AA-DHS cohort may be enriched for novel genomic loci as insulin resistance is associated with decreased vitamin D concentrations [41,42]. Indeed, eight novel loci were identified for their contribution to 25OHD, 1,25(OH)_2_D_3_, and BAVD while two independent loci were replicated for association with VDBP. This study represents the first GWAS to explore the broad range of vitamin D metabolites. As such, additional studies are required to replicate associated loci. With identification of putative functional variants in coding regions, functional studies are needed to validate their role and provide insight into physiology.

## Conclusions

Ethnic-specific differences in bone mineral density and vitamin D metabolism phenotypes could provide insight into the developmental mechanisms of subclinical atherosclerosis and osteoporosis.

## Supporting information

S1 Fig Quantile-Quantile plot for AA-DHS by trait. Each black dot represents an observed statistic [log_10_(P)] (y-axis) versus the corresponding expected statistic (x-axis). The red line denotes the null distribution, which assumes no association. Corresponding inflation factors (λ) are listed for each trait. A. 25-hydroxyvitamin D (λ = 1.01), B. 1,25-dihydroxyvitamin D (λ = 1.01), C. Vitamin D Binding Protein (λ = 1.03), D. Bioavailable Vitamin D (λ = 1.02), E. intact Parathyroid Hormone (λ = 1.01), F. thoracic volumetric bone mineral density (λ = 1.02), G. lumbar volumetric bone mineral density (λ = 1.02).
(DOCX)

S2 Fig Manhattan plots for trait association results in the African American-Diabetes Heart Study (AA-DHS). A. intact Parathyroid Hormone, B. thoracic volumetric bone mineral density, C. lumbar volumetric bone mineral density.
(DOCX)

S3 Fig Regional association plots for variants associated at genome-wide significance (P<5.0x10^-8^) with vitamin D concentrations in the AA-DHS cohort. The -log10(p value) is shown on the left y-axis, recombination rates [expressed in centiMorgans (cM) per Mb; NCBI Build GRCh37; highlighted in blue] are shown on the right y-axis and position in Mb is on the x-axis. Pairwise linkage disequilibrium (r^2^) of each variant with the top variant in the region is indicated by its color. A. rs116788687 (25OHD), B. rs143555701 (25OHD), C. rs116950775 (25OHD), D. rs114001906 (25OHD), E. rs111955953 (25OHD), F. rs117075918 (25OHD), G. rs80068476 (1,25(OH_3_)D_3_) and H. rs7041 (BAVD).
(DOCX)

S1 Table Phenotype correlations among traits examined in AA-DHS. (DOCX)

S2 Table Summary of variants associated (P<5.0x10^-8^) with vitamin D concentrations, parathyroid hormone concentrations, and bone mineral density in the African American-Diabetes Heart Study cohort with additional covariate adjustment for menopause status. (DOCX)

S3 Table Interaction analysis results for rs7041 and rs4588 with VDBP in AA-DHS. (DOCX)

S4 Table Summary of association results in AA-DHS for variants previously reported for association with vitamin D metabolism and bone mineral density. (DOCX)

S5 Table Replication results in IRASFS for variants identified in the AA-DHS. (DOCX)
