# Supplementary material for: Genome-wide association study of vitamin D concentrations and bone mineral density in the African American-Diabetes Heart Study
Source: PLoS One. 2021 May 20;16(5):e0251423. doi: 10.1371/journal.pone.0251423 (PMC8136717; doi:10.1371/journal.pone.0251423)

**Supplementary Figure 1.** Quantile-Quantile plot for AA-DHS by trait. Each black dot represents an observed statistic [log_10_(P)] (y-axis) versus the corresponding expected statistic (x-axis). The red line denotes the null distribution, which assumes no association. Corresponding inflation factors (λ) are listed for each trait. A. 25-hydroxyvitamin D (λ=1.01), B. 1,25-dihydroxyvitamin D (λ=1.01), C. Vitamin D Binding Protein (λ=1.03), D. Bioavailable Vitamin D (λ=1.02), E. intact Parathyroid Hormone (λ=1.01), F. thoracic volumetric bone mineral density (λ=1.02), G. lumbar volumetric bone mineral density (λ=1.02).

1. 25OHD (λ=1.01) B. 1,25(OH)_2_D_3_ (λ=1.01)


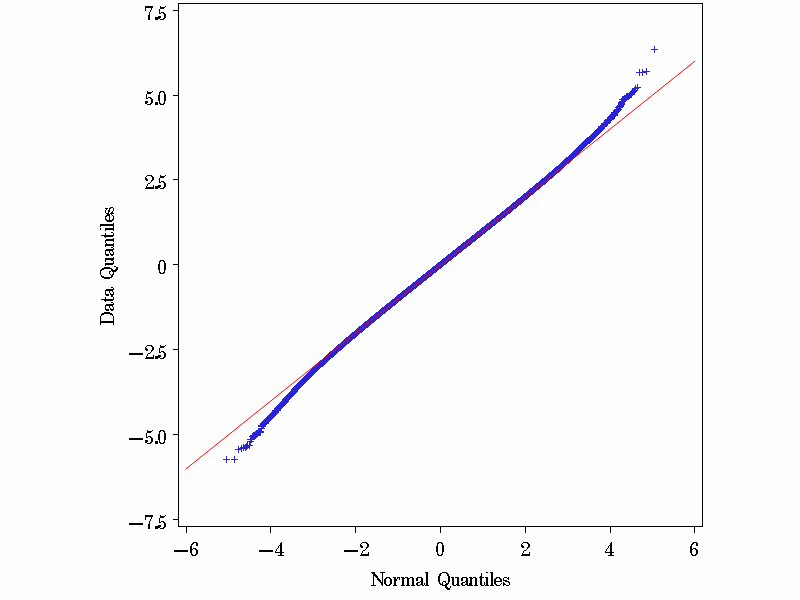

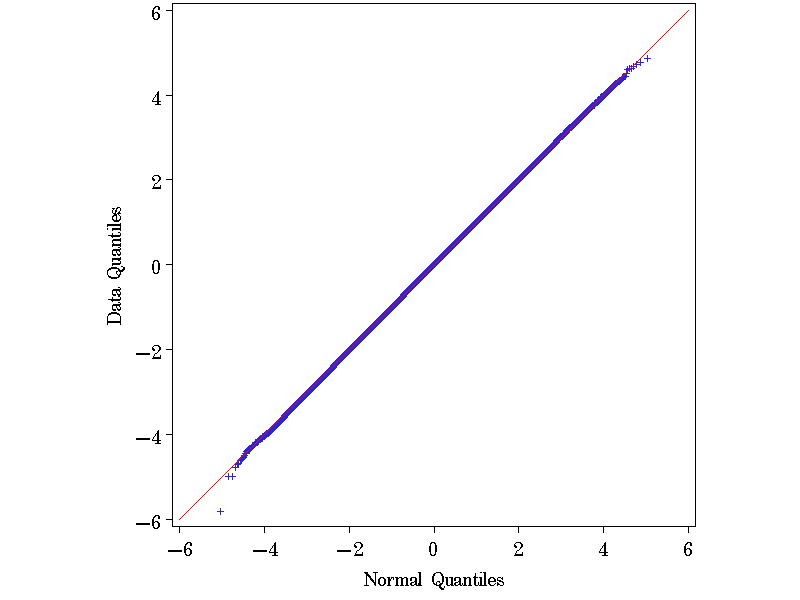


1. VDBP (λ=1.03) D. BAVD (λ=1.02)


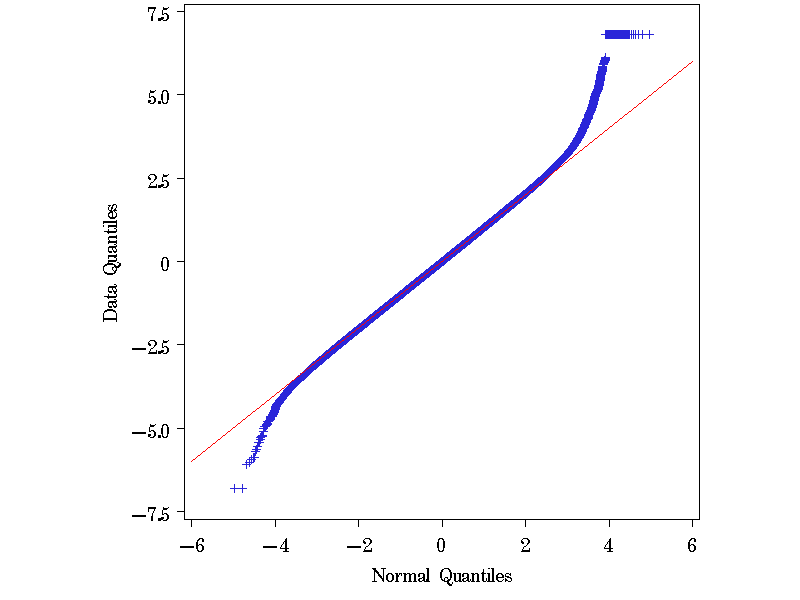

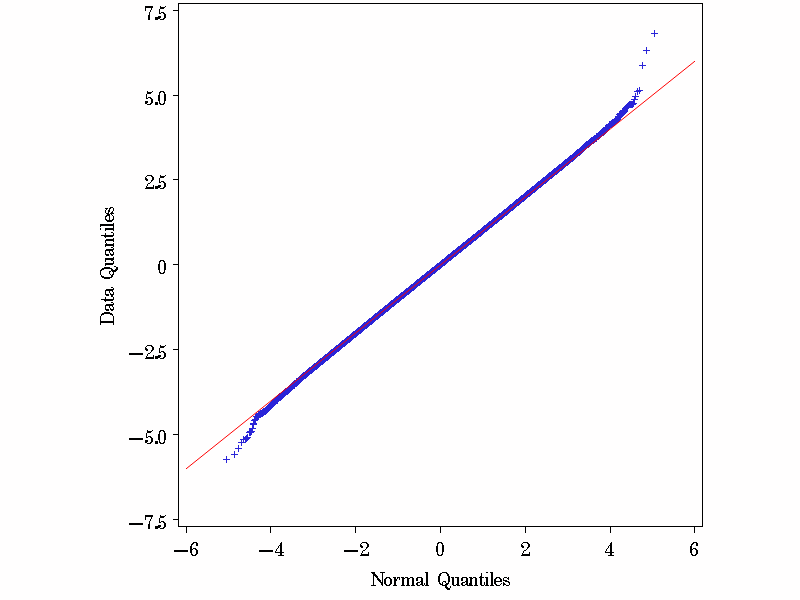


E. iPTH (λ=1.01) F. thoracic vBMD (λ=1.02)


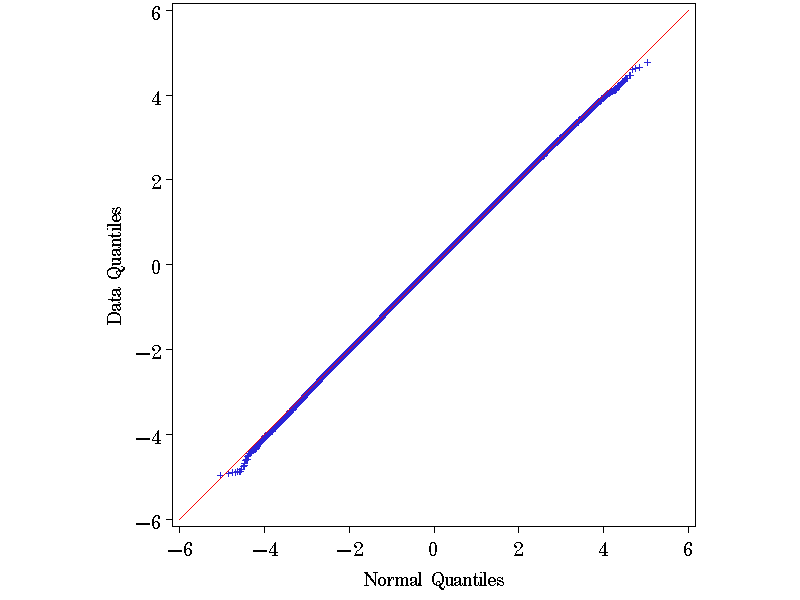

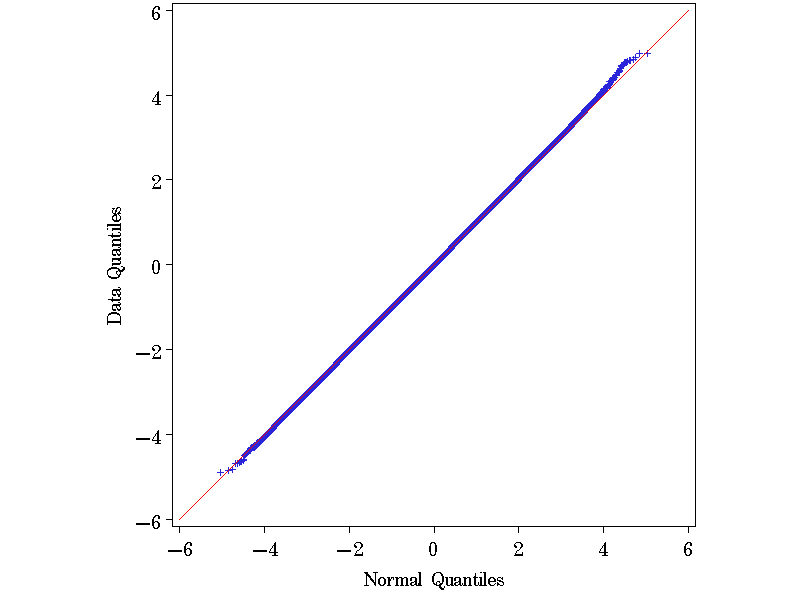


G. lumbar vBMD (λ=1.02)


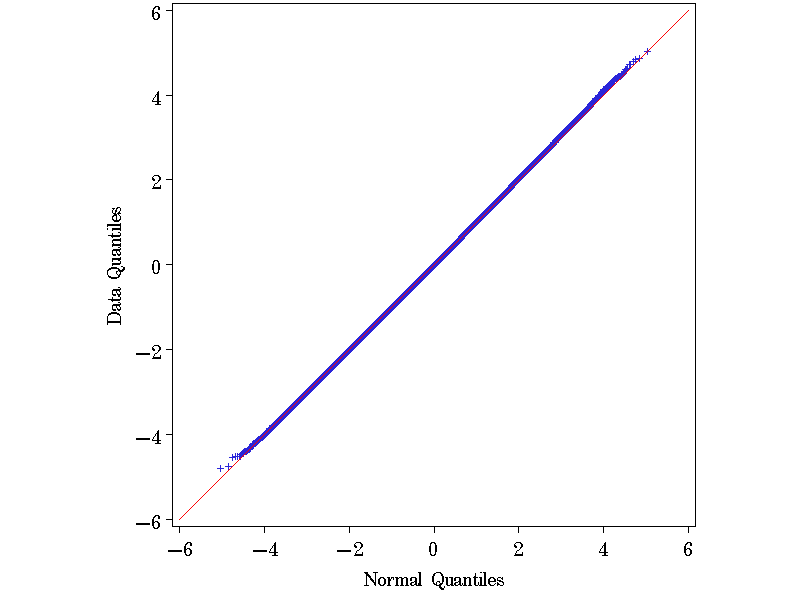

Supplement: S1 Fig — Each black dot represents an observed statistic [log10(P)] (y-axis) versus the corresponding expected statistic (x-axis). The red line denotes the null distribution, which assumes no association. Corresponding inflation factors (λ) are listed for each trait. A. 25-hydroxyvitamin D (λ = 1.01), B. 1,25-dihydroxyvitamin D (λ = 1.01), C. Vitamin D Binding Protein (λ = 1.03), D. Bioavailable Vitamin D (λ = 1.02), E. intact Parathyroid Hormone (λ = 1.01), F. thoracic volumetric bone mineral density (λ = 1.02), G. lumbar volumetric bone mineral density (λ = 1.02). (DOCX) [file pone.0251423.s001.docx]
