# Supplementary material for: Genome-wide association study of vitamin D concentrations and bone mineral density in the African American-Diabetes Heart Study
Source: PLoS One. 2021 May 20;16(5):e0251423. doi: 10.1371/journal.pone.0251423 (PMC8136717; doi:10.1371/journal.pone.0251423)

**Supplementary Figure 2.** Manhattan plots for trait association results in the African American-Diabetes Heart Study (AA-DHS). A. intact Parathyroid Hormone, B. thoracic volumetric bone mineral density, C. lumbar volumetric bone mineral density.

A. iPTH


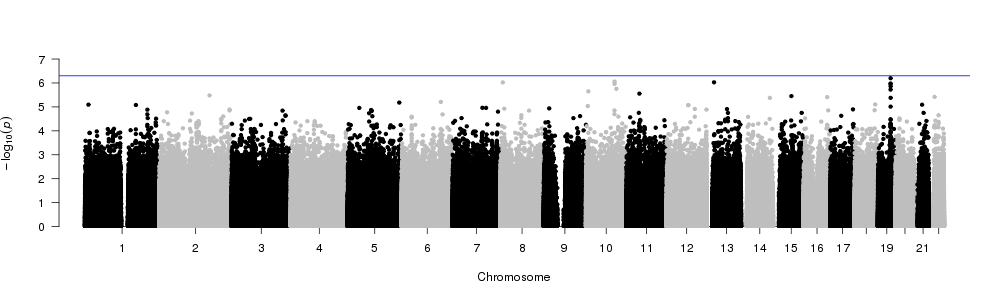


B. thoracic vBMD


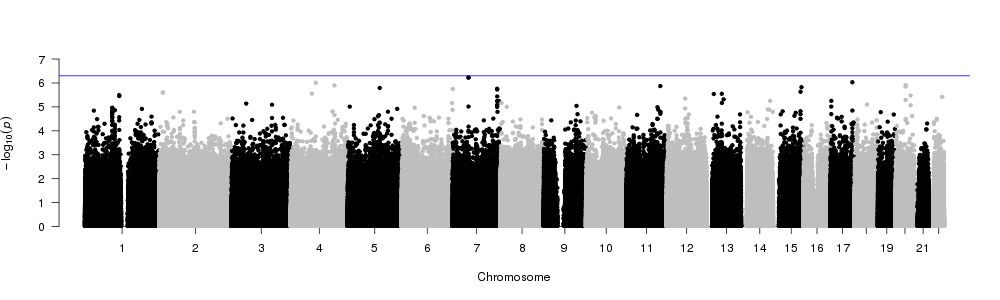


C. lumbar vBMD.


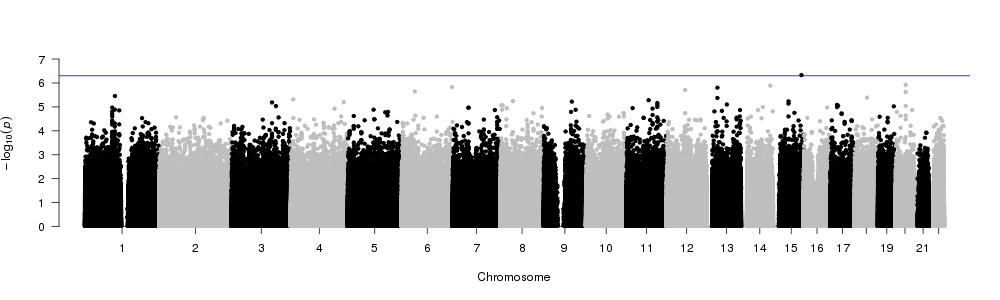

Supplement: S2 Fig — A. intact Parathyroid Hormone, B. thoracic volumetric bone mineral density, C. lumbar volumetric bone mineral density. (DOCX) [file pone.0251423.s002.docx]
