# Supplementary material for: Genome-wide association study of vitamin D concentrations and bone mineral density in the African American-Diabetes Heart Study
Source: PLoS One. 2021 May 20;16(5):e0251423. doi: 10.1371/journal.pone.0251423 (PMC8136717; doi:10.1371/journal.pone.0251423)
Supplement: S3 Fig — The -log10(p value) is shown on the left y-axis, recombination rates [expressed in centiMorgans (cM) per Mb; NCBI Build GRCh37; highlighted in blue] are shown on the right y-axis and position in Mb is on the x-axis. Pairwise linkage disequilibrium (r2) of each variant with the top variant in the region is indicated by its color. A. rs116788687 (25OHD), B. rs143555701 (25OHD), C. rs116950775 (25OHD), D. rs114001906 (25OHD), E. rs111955953 (25OHD), F. rs117075918 (25OHD), G. rs80068476 (1,25(OH3)D3) and H. rs7041 (BAVD). (DOCX) [file pone.0251423.s003.docx]

**Supplementary Figure 3.** Regional association plots for variants associated at genome-wide significance (P<5.0x10^-8^) with vitamin D concentrations in the AA-DHS cohort. The -log10(p value) is shown on the left y-axis, recombination rates [expressed in centiMorgans (cM) per Mb; NCBI Build GRCh37; highlighted in blue] are shown on the right y-axis and position in Mb is on the x-axis. Pairwise linkage disequilibrium (r^2^) of each variant with the top variant in the region is indicated by its color. A. rs116788687 (25OHD), B. rs143555701 (25OHD), C. rs116950775 (25OHD), D. rs114001906 (25OHD), E. rs111955953 (25OHD), F. rs117075918 (25OHD), G. rs80068476 (1,25(OH_3_)D_3_) and H. rs7041 (BAVD).

A. rs116788687 (25OHD) B. rs143555701 (25OHD)


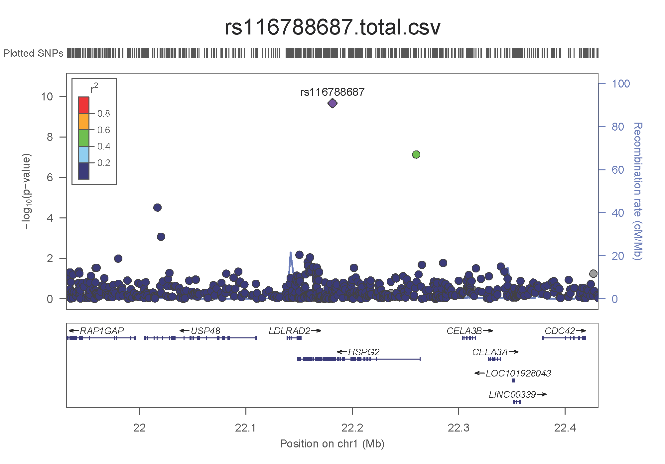

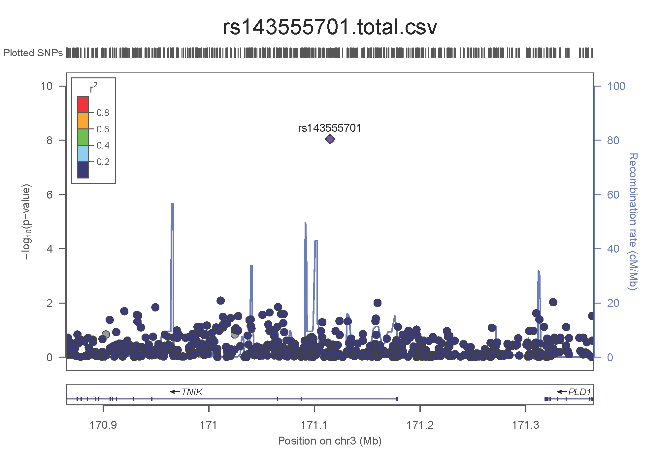


C. rs116950775 (25OHD) D. rs114001906 (25OHD)


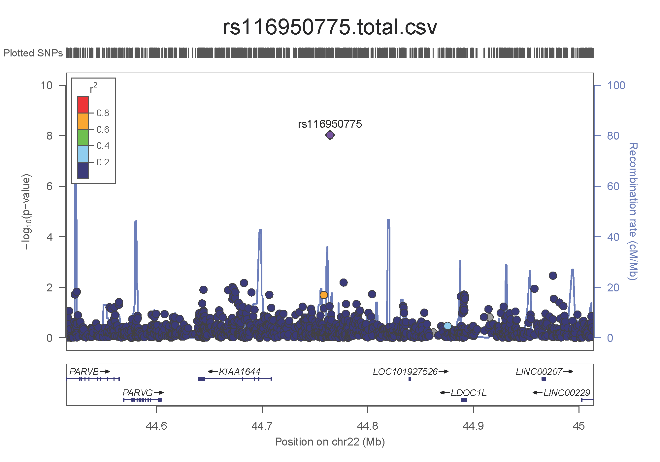

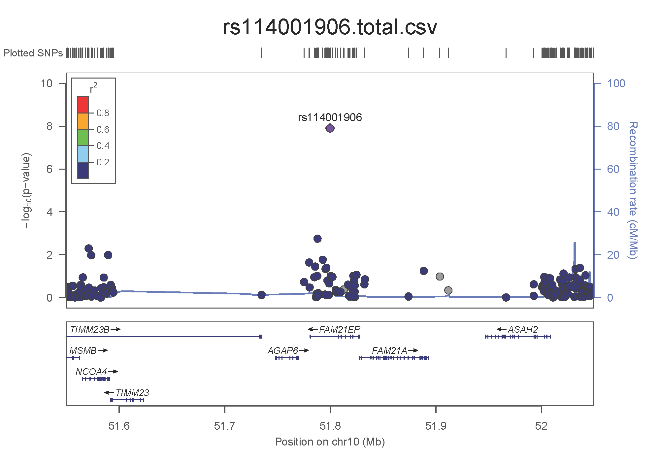


E. rs111955953 (25OHD) F. rs117075918 (25OHD)


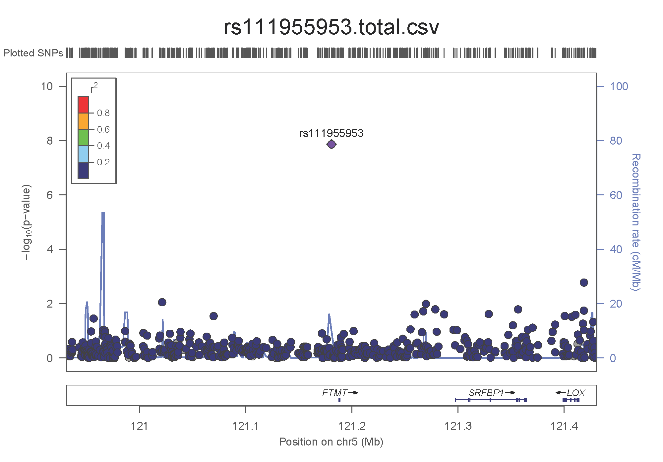

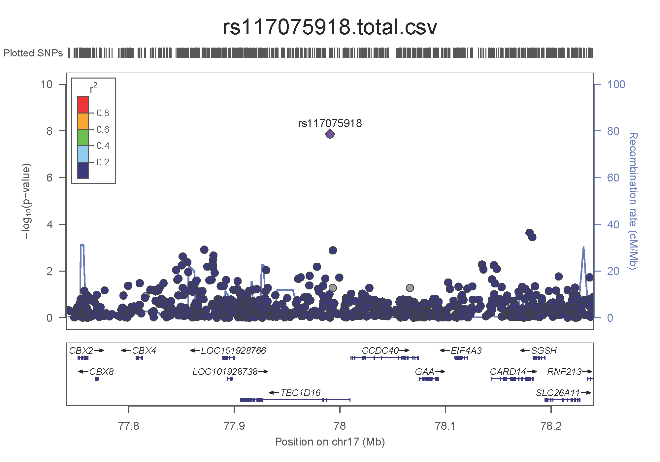


G. rs80068476 (1,25(OH_3_)D_3_) H. rs7041 (BAVD)


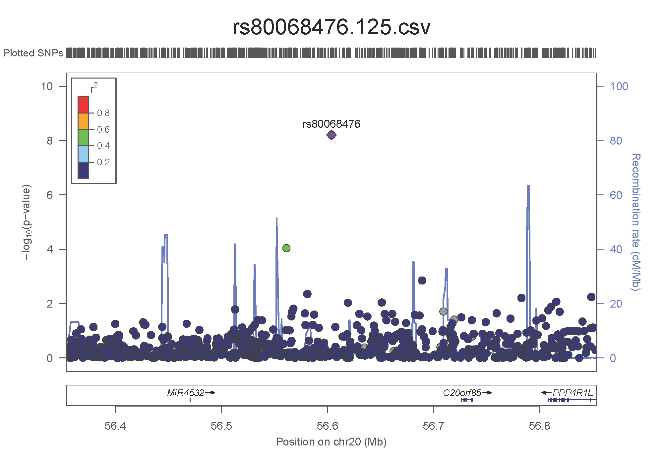
 **
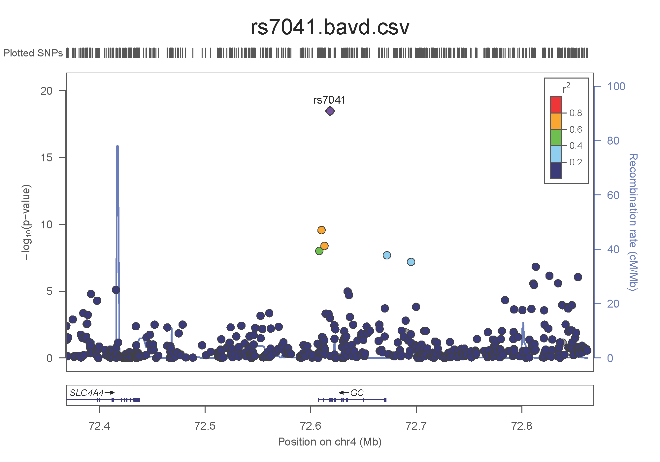
**
