# Supplementary material for: Genome-wide association study of vitamin D concentrations and bone mineral density in the African American-Diabetes Heart Study
Source: PLoS One. 2021 May 20;16(5):e0251423. doi: 10.1371/journal.pone.0251423 (PMC8136717; doi:10.1371/journal.pone.0251423)
Supplement: S1 Table — (DOCX) [file pone.0251423.s004.docx]

**Supplementary Table 1.** Phenotype correlations among traits examined in AA-DHS.

|  | **25OHD** | **1,25(OH)_2_D_3_** | **iPTH** | **BAVD** | **VDBP** | **lumbar vBMD** | **thoracic vBMD** |
| --- | --- | --- | --- | --- | --- | --- | --- |
| **25OHD** | 1 | 0.02 | 0.13 | 0.05 | -0.01 | -0.04 | -0.03 |
| **1,25(OH)_2_D_3_** |  | 1 | -0.2 | 0.78 | 0.03 | -0.11 | -0.09 |
| **iPTH** |  |  | 1 | -0.2 | 0.03 | -0.09 | -0.08 |
| **BAVD** |  |  |  | 1 | -0.52 | -0.07 | -0.07 |
| **VDBP** |  |  |  |  | 1 | -0.01 | 0.01 |
| **lumbar vBMD** |  |  |  |  |  | 1 | 0.92 |
| **thoracic vBMD** |  |  |  |  |  |  | 1 |
