# Supplementary material for: Genome-wide association study of vitamin D concentrations and bone mineral density in the African American-Diabetes Heart Study
Source: PLoS One. 2021 May 20;16(5):e0251423. doi: 10.1371/journal.pone.0251423 (PMC8136717; doi:10.1371/journal.pone.0251423)
Supplement: S2 Table — (DOCX) [file pone.0251423.s005.docx]

**Supplementary Table 2:** Summary of variants associated (P<5.0x10^-8^) with vitamin D concentrations, parathyroid hormone concentrations, and bone mineral density in the African American-Diabetes Heart Study cohort with additional covariate adjustment for menopause status.

| **Variant** | **Position (hg19)** | **Alleles^1^** | **Gene^2^** | **Additive P-value^3^** | **Additive P-value^4^** |
| --- | --- | --- | --- | --- | --- |
| ***25-hydroxyvitamin D*** | |  |  |  |  |
| rs116788687 | chr1:22181360 | G/C | *HSPG2* | 2.17x10^-10^ | 5.06x10^-05^ |
| rs143555701 | chr3:171114695 | T/G | *TNIK* | 8.98x10^-09^ | 1.03x10^-08^ |
| rs116950775 | chr22:44764343 | T/C | *KIAA1644/LDOC1L* | 9.29x10^-09^ | 0.31 |
| rs114001906 | chr10:51799688 | C/T | *FLJ31813* | 1.25x10^-08^ | 3.62x10^-09^ |
| rs111955953 | chr5:121180672 | C/A | *-/FTMT* | 1.36x10^-08^ | 1.83x10^-08^ |
| rs117075918 | chr17:77990613 | C/T | *TBC1D16* | 1.40x10^-08^ | 4.52x10^-08^ |
| ***1,25-dihydroxyvitamin D*** | |  |  |  |  |
| rs80068476 | chr20:56603691 | T/C | *-/C20orf85* | 6.19x10^-09^ | 1.32x10^-08^ |
| ***Vitamin D Binding Protein*** | |  |  |  |  |
| rs7041 | chr4:72618334 | C/A | *GC* | 9.35x10^-86^ | 9.29x10^-127^ |
| ***Vitamin D Binding Protein (adjusted for GC rs7041)*** | | | | | |
| rs4588 | chr4:72618323 | T/G | *GC* | 1.43x10^-14^ | 1.43x10^-16^ |
| rs1155563 | chr4:72643488 | C/T | *GC* | 2.22x10^-11^ | 1.41x10^-11^ |
| rs221999 | chr4:72649048 | G/A | *GC* | 2.23x10^-09^ | 1.76x10^-09^ |
| rs3755967 | chr4:72609398 | T/C | *GC* | 1.15x10^-08^ | 1.03x10^-08^ |
| rs2282679 | chr4:72608383 | G/T | *GC* | 2.03x10^-08^ | 2.10x10^-08^ |
| rs9016 | chr4:72618296 | T/C | *GC* | 3.52x10^-08^ | 1.89x10^-08^ |
| ***Bioavailable Vitamin D*** | |  |  |  |  |
| rs7041 | chr4:72618334 | C/A | *GC* | 3.30x10^-19^ | 3.19x10^-19^ |
| rs222047 | chr4:72610208 | C/A | *GC* | 2.68x10^-10^ | 2.37x10^-10^ |
| rs705119 | chr4:72613036 | C/A | *GC* | 4.09x10^-09^ | 2.22x10^-09^ |
| rs705117 | chr4:72608115 | T/C | *GC* | 9.67x10^-09^ | 1.33x10^-08^ |
| rs11939173 | chr4:72672158 | A/G | *GC/NPFFR2* | 2.05x10^-08^ | 2.31x10^-08^ |

^1^Reference/other allele, ^2^nearest annotated gene within 500kb, ^3^Covariates: age, sex, body mass index (BMI), kidney function based on the Chronic Kidney Disease-Epidemiology Collaboration (CKD-EPI) estimated glomerular filtration rate (eGFR), multivitamin use, and global African ancestry proportions, ^4^Covariates: age, sex as a three level variable to account for menopause status, body mass index (BMI), kidney function based on the Chronic Kidney Disease-Epidemiology Collaboration (CKD-EPI) estimated glomerular filtration rate (eGFR), multivitamin use, and global African ancestry proportions. *Note: Results with inclusion of sex as a three level variable to account for menopause status should be viewed with caution for 25-hydroxyvitamin D and 1,25-dihydroxyvitamin D owing to reduced samples sizes.*
