# Supplementary material for: Genome-wide association study of vitamin D concentrations and bone mineral density in the African American-Diabetes Heart Study
Source: PLoS One. 2021 May 20;16(5):e0251423. doi: 10.1371/journal.pone.0251423 (PMC8136717; doi:10.1371/journal.pone.0251423)
Supplement: S3 Table — (DOCX) [file pone.0251423.s006.docx]

**Supplementary Table 3.** Interaction analysis results for rs7041 and rs4588 with VDBP in AA-DHS.

|  |  |  | **VDBP** |  |
| --- | --- | --- | --- | --- |
| **rs7041^1^** | **rs4588^2^** | **N** | **Mean** | **SD** |
| AA | GG | 290 | 43.38 | 22.14 |
| AA | GT | 83 | 68.38 | 27.78 |
| AA | TT | 6 | 100.20 | 49.42 |
| AC | GG | 112 | 148.46 | 57.67 |
| AC | GT | 14 | 161.92 | 76.54 |
| AC | TT | 0 | - | - |
| CC | GG | 17 | 271.87 | 92.57 |
| CC | GT | 0 | - | - |
| CC | TT | 0 | - | - |

^1^rs7041-A was associated with decreased levels of VDBP in single SNP analyses, ^2^rs4588-G was associated with decreased levels of VDBP in single SNP analyses.
