# Supplementary material for: Genome-wide association study of vitamin D concentrations and bone mineral density in the African American-Diabetes Heart Study
Source: PLoS One. 2021 May 20;16(5):e0251423. doi: 10.1371/journal.pone.0251423 (PMC8136717; doi:10.1371/journal.pone.0251423)
Supplement: S4 Table — (DOCX) [file pone.0251423.s007.docx]

**Supplementary Table 4:** Summary of association results in AA-DHS for variants previously reported for association with vitamin D metabolism and bone mineral density.

| **Variant** | **Alleles^1^** | **Gene^2^** | **Freq^3^** | **Beta ± SE^3^** | **P-value^3^** | **MAF^4^** | **Beta ± SE^4^** | **P-value^4^** |
| --- | --- | --- | --- | --- | --- | --- | --- | --- |
| ***25-hydroxyvitamin D***^5^ | | | | | | | | |
| rs3755967 | T/C | *GC* | 0.28 | -0.089 ± 0.0023 | 4.74E-343 | 0.096 | -0.080 ± 0.060 | 0.14 |
| rs12785878^9^ | T/G | *NADSYN/DHCR7* | 0.75 | 0.036 ± 0.0022 | 3.80E-62 | 0.27 | -0.026 ± 0.037 | 0.48 |
| rs10741657^9^ | A/G | *CYP2R1* | 0.40 | 0.031 ± 0.0022 | 2.05E-46 | 0.35 | 0.061 ± 0.036 | 0.086 |
| rs17216707 | T/C | *CYP24A1* | 0.79 | 0.026 ± 0.0027 | 8.14E-23 | 0.055 | -0.050 ± 0.070 | 0.49 |
| rs10745742^9^ | T/C | *AMDHD1* | 0.40 | 0.017 ± 0.0022 | 1.88E-14 | 0.35 | -0.027 ± 0.033 | 0.42 |
| rs8018720 | C/G | *SEC23A* | 0.82 | -0.017 ± 0.0029 | 4.72E-09 | 0.14 | -0.070 ± 0.050 | 0.15 |
| ***Vitamin D Binding Protein*^6^** | | | | | | | | |
| rs7041 | T/G | *GC* | 0.35 | -2109.34 ± 63.35 | 1.42E-246 | 0.15 | -0.44 ± 0.020 | 9.35E-86 |
| rs705117 | G/A | *GC* | 0.13 | -2026.78 ± 100.91 | 4.68E-91 | 0.34 | -0.28 ± 0.019 | 4.58E-43 |
| rs12144344 | T/C | *ST6GALNAC3* | 0.44 | 396.39 ± 80.21 | 5.93E-07 | 0.18 | 0.013 ± 0.027 | 0.62 |
| rs6684432 | T/C | *GALNT2* | 0.16 | 457.73 ± 109.81 | 1.36E-05 | 0.045 | -0.040 ± 0.048 | 0.40 |
| ***intact Parathyroid Hormone*^7^** | | | | | | | | |
| rs6127099^9^ | T/A | CYP24A1 | 0.34 | 0.07 ± 0.005 | 2.2E-43 | 0.19 | -0.075 ± 0.038 | 0.049 |
| rs4074995 | G/A | RGS14 | 0.71 | 0.03 ± 0.005 | 7.7E-12 | 0.10 | 0.046 ± 0.050 | 0.36 |
| rs219779^9^ | G/A | CLDN14 | 0.75 | 0.03 ± 0.005 | 1.6E-11 | 0.32 | 0.078 ± 0.031 | 0.012 |
| rs4443100^9^ | G/C | RTDR1 | 0.32 | 0.02 ± 0.005 | 3.0E-07 | 0.18 | -0.009 ± 0.037 | 0.82 |
| rs73186030^9^ | T/C | CASR | 0.14 | 0.03 ± 0.006 | 6.4E-07 | - | - | - |
| ***Lumbar vBMD*^8^** | | | | | | | | |
| rs17482952^9^ | A/G | *WLS* | 0.93 | 0.07 | 1.69E‐08 | 0.083 | -0.036 ± 0.16 | 0.82 |
| rs12407028 | T/C | *WLS* | 0.6 | 0.08 | 3.11E‐45 | 0.27 | 0.071 ± 0.10 | 0.48 |
| rs7521902^9^ | A/C | *WNT4* | 0.31 | ‐0.05 | 9.66E‐11 | 0.17 | 0.21 ± 0.12 | 0.078 |
| rs6426749^9^ | C/G | *ZBTB40* | 0.17 | 0.1 | 1.86E‐44 | 0.25 | 0.14 ± 0.10 | 0.18 |
| rs4233949^9^ | C/G | *SPTBN1* | 0.38 | 0.05 | 2.25E‐18 | 0.32 | 0.061 ± 0.093 | 0.51 |
| rs1878526 | A/G | *INSIG2* | 0.22 | 0.04 | 1.22E‐10 | 0.32 | 0.14 ± 0.092 | 0.14 |
| rs1346004^9^ | A/G | *GALNT3* | 0.5 | ‐0.06 | 3.87E‐30 | 0.23 | -0.022 ± 0.099 | 0.82 |
| rs430727 | T/C | *CTNNB1* | 0.48 | ‐0.05 | 1.54E‐18 | 0.38 | -0.081 ± 0.090 | 0.37 |
| rs344081 | T/C | *LEKR1* | 0.87 | 0.06 | 4.46E‐12 | 0.49 | 0.20 ± 0.088 | 0.021 |
| rs3755955 | A/G | *IDUA* | 0.16 | ‐0.06 | 5.24E‐15 | 0.10 | 0.093 ± 0.14 | 0.52 |
| rs6532023^9^ | T/G | *MEPE* | 0.34 | 0.06 | 1.23E‐27 | 0.34 | 0.042 ± 0.093 | 0.65 |
| rs11755164^9^ | T/C | *SUPT3H* | 0.4 | ‐0.04 | 5.60E‐11 | 0.45 | -0.069 ± 0.087 | 0.43 |
| rs9466056 | A/G | *CDKAL1* | 0.38 | ‐0.03 | 3.56E‐08 | 0.37 | -0.21 ± 0.087 | 0.016 |
| rs13204965 | A/C | *RSPO3* | 0.76 | 0.04 | 3.61E‐10 | 0.052 | 0.14 ± 0.20 | 0.49 |
| rs7751941 | A/G | *C6orf97* | 0.21 | ‐0.08 | 1.99E‐24 | 0.19 | -0.030 ± 0.11 | 0.77 |
| rs4869742^9^ | T/C | *C6orf97* | 0.31 | ‐0.08 | 3.95E‐35 | 0.32 | -0.082 ± 0.090 | 0.36 |
| rs10226308^9^ | A/G | *TXNDC3* | 0.84 | ‐0.06 | 6.40E‐13 | 0.074 | -0.088 ± 0.16 | 0.58 |
| rs6959212 | T/C | *STARD3NL* | 0.32 | ‐0.07 | 3.76E‐38 | 0.36 | -0.19 ± 0.088 | 0.030 |
| rs4727338^9^ | C/G | *SLC25A13* | 0.67 | 0.07 | 2.13E‐35 | 0.20 | 0.11 ± 0.11 | 0.34 |
| rs3801387 | A/G | *WNT16* | 0.74 | ‐0.09 | 3.17E‐51 | 0.36 | 0.067 ± 0.090 | 0.46 |
| rs13245690 | A/G | *C7orf58* | 0.65 | 0.05 | 1.65E‐11 | 0.24 | 0.035 ± 0.097 | 0.72 |
| rs2062377^9^ | A/T | *TNFRSF11B* | 0.57 | ‐0.08 | 3.16E‐39 | 0.14 | -0.098 ± 0.12 | 0.43 |
| rs3905706 | T/C | *MPP7* | 0.22 | 0.05 | 2.41E‐16 | 0.42 | 0.082 ± 0.090 | 0.37 |
| rs1373004^9^ | T/G | *MBL2* | 0.13 | ‐0.06 | 1.56E‐12 | 0.45 | -0.046 ± 0.086 | 0.60 |
| rs7071206 | T/C | *KCNMA1* | 0.78 | ‐0.06 | 5.02E‐19 | 0.15 | -0.14 ± 0.12 | 0.23 |
| rs7932354^9^ | T/C | *ARHGAP1* | 0.31 | 0.04 | 5.45E‐12 | 0.21 | 0.22 ± 0.11 | 0.057 |
| rs10835187^9^ | T/C | *LIN7C* | 0.55 | ‐0.03 | 4.90E‐08 | 0.16 | 0.054 ± 0.13 | 0.67 |
| rs163879 | T/C | *DCDC5* | 0.68 | ‐0.04 | 2.19E‐11 | 0.47 | -0.044 ± 0.086 | 0.61 |
| rs3736228 | T/C | *LRP5* | 0.16 | ‐0.08 | 2.08E‐26 | 0.046 | 0.20 ± 0.20 | 0.32 |
| rs2887571 | A/G | *ERC1* | 0.76 | ‐0.04 | 5.59E‐12 | 0.19 | 0.20 ± 0.11 | 0.077 |
| rs12821008 | T/C | *DHH* | 0.39 | 0.05 | 1.17E‐15 | - | - | - |
| rs736825^9^ | C/G | *HOXC6* | 0.56 | 0.05 | 7.68E‐16 | 0.26 | 0.13 ± 0.10 | 0.19 |
| rs2016266 | A/G | *SP7* | 0.68 | ‐0.05 | 2.95E‐20 | 0.43 | -0.11 ± 0.088 | 0.20 |
| rs9533090^9^ | T/C | *AKAP11* | 0.49 | ‐0.10 | 4.82E‐68 | - | - | - |
| rs1286083 | T/C | *RPS6KA5* | 0.81 | ‐0.05 | 1.75E‐14 | 0.26 | -0.018 ± 0.099 | 0.86 |
| rs11623869 | T/G | *MARK3* | 0.35 | ‐0.04 | 5.12E‐11 | 0.20 | -0.11 ± 0.11 | 0.33 |
| rs4985155 | A/G | *NTAN1* | 0.67 | ‐0.03 | 2.15E‐09 | 0.35 | 0.057 ± 0.090 | 0.53 |
| rs9921222 | T/C | *AXIN1* | 0.48 | ‐0.04 | 1.00E‐16 | 0.31 | 0.018 ± 0.095 | 0.85 |
| rs13336428 | A/G | *C16orf38* | 0.43 | ‐0.04 | 1.66E‐13 | 0.21 | -0.0085 ± 0.11 | 0.94 |
| rs1564981 | A/G | *CYLD* | 0.5 | ‐0.04 | 1.95E‐10 | 0.14 | -0.042 ± 0.12 | 0.73 |
| rs10048146 | A/G | *FOXL1* | 0.8 | 0.05 | 3.09E‐11 | 0.13 | 0.064 ± 0.12 | 0.60 |
| rs4790881 | A/C | *SMG6* | 0.69 | 0.03 | 3.38E‐09 | 0.12 | -0.0077 ± 0.13 | 0.95 |
| rs4792909 | T/G | *SOST* | 0.37 | 0.04 | 9.43E‐10 | 0.27 | -0.017 ± 0.10 | 0.86 |
| rs227584 | A/C | *C17orf53* | 0.7 | ‐0.04 | 9.92E‐10 | 0.18 | 0.0025 ± 0.12 | 0.98 |
| rs1864325^9^ | T/C | *MAPT* | 0.22 | ‐0.04 | 4.89E‐11 | - | - | - |
| rs884205 | A/C | *TNFRSF11A* | 0.27 | ‐0.05 | 1.58E‐17 | 0.053 | 0.15 ± 0.19 | 0.43 |
| rs10416218^9^ | T/C | *GPATCH1* | 0.73 | ‐0.04 | 6.64E‐11 | 0.26 | -0.16 ± 0.10 | 0.12 |
| rs3790160^9^ | T/C | *JAG1* | 0.5 | 0.05 | 3.07E‐19 | 0.42 | -0.070 ± 0.091 | 0.44 |

^1^effect allele/other allele from previous publication, ^2^nearest annotated gene within 500kb, ^3^Previously reported association statistics (where available), ^4^AA-DHS association statistics, ^5^Nat Commun. 2018 Jan 17;9(1):260., ^6^Am J Clin Nutr. 2014 Jun;99(6):1424-31., ^7^J Am Soc Nephrol. 2017 May;28(5):1553-1565., ^8^Nat Genet. 2012 Apr 15;44(5):491-5, ^9^Variants were imputed from 1000 Genomes,
