# Supplementary material for: Genome-wide association study of vitamin D concentrations and bone mineral density in the African American-Diabetes Heart Study
Source: PLoS One. 2021 May 20;16(5):e0251423. doi: 10.1371/journal.pone.0251423 (PMC8136717; doi:10.1371/journal.pone.0251423)
Supplement: S5 Table — (DOCX) [file pone.0251423.s008.docx]

**Supplementary Table 5**. Replication results in IRASFS for variants identified in the AA-DHS.

| Variant | Position (hg19) | Alleles | MAF | Gene | Beta ± SE | Additive P-value |
| --- | --- | --- | --- | --- | --- | --- |
| ***25-hydroxyvitamin D*** | | | | | | |
| rs116788687 | chr1:22181360 | C/G | 0.0046 | *HSPG2* | 0.040 ± 0.41 | 0.92 |
| rs143555701 | chr3:171114695 | T/G | 0.00022 | *TNIK* | -8.92 ± 11.85 | 0.45 |
| rs116950775 | chr22:44764343 | T/C | 0.0068 | *KIAA1644/LDOC1L* | 0.13 ± 0.33 | 0.70 |
| rs114001906 | chr10:51799688 | C/T | 0.0046 | *FLJ31813* | -0.35 ± 0.48 | 0.46 |
| rs111955953 | chr5:121180672 | C/A | 0.023 | *-/FTMT* | -0.13 ± 0.19 | 0.49 |
| rs117075918 | chr17:77990613 | C/T | 0.0026 | *TBC1D16* | 0.14 ± 0.58 | 0.81 |
| ***1,25 di-hydroxyvitamin D*** | | | | | | |
| rs80068476 | chr20:56603691 | T/C | 0.0011 | *-/C20orf85* | -2.08 ± 2.24 | 0.35 |
